# The Rational Treatment of Pulmonary Hemorrhage

**Published:** 1907-11

**Authors:** 


### THE RATIONAL TREATMENT OF PULMONARY HEMORRHAGE.

W. J. Robinson summarizes his paper as follows:

Relieve the patient's intense anxiety by a few kind and encouraging words; unloosen or remove his clothing, and put him in a semi-recumbent position. Give an inhalation of amyl nitrite. Or inject a quarter of a grain of morphine combined with 1:150 to 1.120 grain of atropine. Give 5 to 15 min. of sol. of adrenalin. You may also give a teaspoonful of common salt, dry on the tongue, or 20 to 60 minims of aromatic sulphuric acid, diluted with a small quantity of water. Order an ice-bag on the chest.

If the above measures fail to check the hemorrhage within a short time—half an hour or so—you must cord the extremities; not too tight, but sufficient to prevent the return of the venous blood. Do not under any circumstances give ergot, or alum, gallic and tanic acids, or any other local astringents. The first has no effect as a hemostatic except indirectly in uterine hemorrhage, and by raising the blood pressure in the pulmonary circuit hinders thrombosis. The local astringents put into the stomach can have no effect on the bleeding vessels in the lung, and are injurious by irritating the stomach, causing nausea and vomiting and inducing constipation. Insist upon absolute mental and physical rest, upon a scanty, nutritious and chiefly fluid diet, and relieve constipation either by Epsom salts or by enemata. As a prophylactic against further hemorrhages, you may perhaps have the patient consume large amounts of gelatin, prepared in various forms. Mild degrees of collapse are to be left alone; in severe collapse, administer camphor (hypodermically) and nitroglycerin; also strychnine (do not give digitalis.) Besides, several hot-water bottles are to be applied to the lower extremities. It sometimes becomes necessary to resort to enterocylsis of large amounts of saline solution; or the latter may have to be injected subcutaneously or intravenously. —Therapeutic Medicine, Sept., 1907.

